# Genome-Wide Identification and Bioinformatics Analysis of Thaumatin-like Proteins in *Aegiceras corniculatum*

**DOI:** 10.3390/genes17091023

**Published:** 2026-08-27

**Authors:** Jinchang Xie, Jingwei Pang, Huishao Shi, Xuechao Chen, Junjian Wang, Li Xu, Wei Zhang

**Affiliations:** 1 College of Marine Science, Beibu Gulf University, Qinzhou 535011, China; 2 Fourth Institute of Oceanography, Ministry of Natural Resources, Beihai 536000, China

**Keywords:** *Aegiceras corniculatum*, thaumatin-like proteins, bioinformatic analyses, antifungal peptide

## Abstract

Background: The mangrove ecosystem serves as a vital coastal ecotone, providing essential ecological services, such as water purification, shoreline protection, and biodiversity maintenance. Fungal pathogens threaten mangrove health and contribute to ecosystem degradation, but the molecular mechanisms underlying disease resistance in mangroves remain poorly explored. Introdustion: Thaumatin-like proteins (TLPs), belonging to the pathogenesis-related-5 (PR-5) family, play a crucial role in antifungal defense in plants. Method and results: In this study, we identified 23 TLP family members in the mangrove *Aegiceras corniculatum*. The genes encoding TLP family members were unevenly distributed on chromosomes. Collinearity analyses showed that TLP family members in *A. corniculatum* underwent multiple gene duplication events, and Ka/Ks calculations revealed that these duplicated genes were predominantly under purifying selection. Among the 23 TLPs, *Ac*TLP19 was significantly upregulated after *Botrytis cinerea* infection. Subcellular localization prediction and experiments revealed the extracellular localization of *Ac*TLP19. Conclusions: Heterologous expression and antibacterial tests showed that recombinant *Ac*TLP19 had no direct antifungal activity against *B. cinerea* or *Fusarium oxysporum* under the tested conditions, leaving open the possibility that it contributes to mangrove defense through indirect mechanisms, or that its antifungal activity was not captured under the specific assay conditions. This study advances our knowledge of mangrove stress responses and may contribute to future conservation strategies.

## 1. Introduction

Tropical and subtropical regions harbor diverse ecosystems, among which mangrove forests are the most distinctive [1,2]. Functioning as a critical ecotone between terrestrial and marine environments, mangrove ecosystems maintain ecological balance and are among the most biodiverse and productive ecosystems on Earth [3,4]. They are essential for sustaining tropical and subtropical ecological stability and serve as key players in climate change mitigation [5,6].

However, fungal diseases threaten mangrove ecosystems, garnering global concern. For example, in 1982, a top-dieback disease outbreak in The Gambia caused 60% mortality of local mangrove vegetation [7]. In 2000, Andrew et al. [8] documented severe *Cytospora* infections in *Rhizophora mangle* in Puerto Rico, with a disease incidence of 33%. Similarly, Kathiresan [1] reported that approximately 12% of mangrove leaves in Pichavaram, India, were affected by fungal pathogens.

Mangrove plants inhibit fungal invasion by regulating the salt distribution and content in their leaves through three salt metabolism methods [9]. Mangrove plants are rich in secondary metabolites, such as sterols, alkaloids, tannins, flavonoids, naphthoquinones, glycosides, and other terpenoids, which form a unique chemical defense barrier [10,11]. In addition, symbiotic flora play an important protective role for mangrove plants [12,13]. Although host defense peptides (HDPs) are common barriers that evolved in plants to resist microbial stress, research on HDPs is still insufficient in mangrove plants.

The thaumatin-like protein (TLP) is named for its amino acid sequence, which is highly homologous to the sweet-tasting thaumatin protein found in the West African plant *Thaumatococcus danielli* [14]. Despite this high homology, their functions are entirely distinct. The thaumatin protein imparts a sweet taste, and TLP lacks sweetness and exhibits antifungal activity. It belongs to the fifth family of pathogenesis-related (PR) proteins [15,16]. Extensive studies have demonstrated that TLPs play pivotal roles in plant defense against fungal pathogens across diverse species. In *Vitis vinifera*, *Vv*TLP1 and *Vv*TLP29 are significantly upregulated upon *B. cinerea* infection and exhibit direct antifungal activity in vitro [17,18]. Similarly, TLPs in *Triticum aestivum* and *Musa* spp. have been functionally linked to resistance against *Rhizoctonia cerealis* and *F. oxysporum*, respectively [19,20,21]. Genome-wide identification of TLP families in model and crop plants, including *Arabidopsis thaliana*, *Oryza sativa*, and *Populus trichocarpa*, has further revealed their complex evolutionary patterns, including frequent gene duplication and functional divergence under biotic stress [22,23].

However, despite the ecological importance of mangrove ecosystems, research on TLP-mediated defense mechanisms in mangroves remains notably scarce. While chemical barriers and symbiotic microbiomes have been proposed as key protective strategies in mangrove plants [24,25], the molecular basis of their intrinsic immune responses is poorly understood. *A. corniculatum*, a dominant pioneer species in Indo-West Pacific mangroves, frequently suffers from fungal infections in its natural habitat [26], yet no systematic characterization of its TLP gene family has been reported to date. This lack of genomic and functional insight represents a critical knowledge gap that limits our comprehensive understanding of how mangroves cope with fungal pathogens at the molecular level.

In this study, we identified and characterized the TLP family in *A. corniculatum*, analyzing their genomic organization, gene duplication patterns, promoter cis-elements, protein properties, and phylogenetic relationships. Expression profiling revealed significant *Ac*TLP19 upregulation after fungal infection. These findings advance our understanding of innate immunity in mangroves and provide a molecular foundation for developing novel biocontrol strategies against mangrove fungal diseases, offering applications for sustainable coastal ecosystem management.

## 2. Materials and Methods

### 2.1. Thaumatin-like Protein Family Gene Identification in A. corniculatum

Genome sequence data for *A. corniculatum* were retrieved from the China National GeneBank (CNGB) under accession number CNA0017738 [26]. To identify potential TLP family members, we employed a Pfam-derived hidden Markov model (HMM) profile for the thaumatin domain (PF00314) as a search query and conducted homology-based screening against the *A. corniculatum* proteome using HMMER (version 3.3.2). Candidate sequences were retained when they met an E-value threshold ≤ 1 × 10^−5^ and a domain coverage ≥ 50% compared to the full-length PF00314 consensus. Candidate protein sequences were analyzed for the presence of full-length thaumatin domains using the NCBI Batch CD-Search tool (https://www.ncbi.nlm.nih.gov/Structure/cdd/cdd.shtml, accessed on 3 November 2025) with a default E-value cutoff (≤1 × 10^−2^). For candidates exceeding 400 amino acids, the full-length protein sequences were submitted to CD-search (https://www.ncbi.nlm.nih.gov/Structure/cdd/wrpsb.cgi, accessed on 5 November 2025) to detect additional N-terminal functional domains.

### 2.2. Physicochemical Characterization and Chromosomal Location

The physicochemical characteristics of the identified TLP family members were computed using the ProtParam tool (https://web.expasy.org/protparam/, accessed on 3 November 2025). The genomic coordinates of these genes were extracted from the official *A. corniculatum* gene annotation files [26], and their chromosomal distributions were visualized in MapChart (V2.3.2) [27].

### 2.3. Phylogenetic, Gene Structure, Motif, and Promoter Region Analyses and Gene Duplication

A phylogenetic tree was inferred using the neighbor-joining algorithm implemented in MEGA11, with bootstrap support assessed over 1000 replicates. The analysis included TLP family members from *A. corniculatum*, *Avicennia marina*, *V. vinifera*, *A. thaliana*, and *P. trichocarpa* [14,17,22]. The resulting tree was visualized and annotated using EVOLVIEW (V2.0, https://www.evolgenius.info/evolview, accessed on 3 November 2025). Gene structural features, including exon–intron organization, were examined using the Gene Structure Display Server (GSDS2.0; http://gsds.cbi.pku.edu.cn, accessed on 5 April 2025). Conserved sequence motifs were identified de novo using the MEME Suite(Version 5.5.9) (http://meme-suite.org/, accessed on 5 November 2025). Putative cis-regulatory elements in the 1.5 kb promoter regions upstream of each gene were predicted using PlantCARE (V2.0, http://sphinx.rug.ac.be:8080/PlantCARE/, accessed on 6 November 2025), and graphical representations of the gene structures, motifs, and regulatory elements were generated using TBtools (V2.0) [28]. The occurrence of TLP family members in *A. corniculatum* and their duplication events were analyzed and visualized using MCScanX (V.2.0, University of Georgia, Athens, GA, USA). Non-synonymous (ka) and synonymous (ks) substitution rates for each duplicated TLP family member gene in *A. corniculatum* were calculated using KaKs_Calculator 2.0.

### 2.4. Fungal Culture, Plant Culture, and Infection

*B. cinerea* (CGMCC 3.3789) and *F. oxysporum* (SHBCC D11648, AS3.1789) were purchased from the Shanghai Preservation Microorganisms Center (Shanghai, China). Potato dextrose agar (PDA) medium was used for activation and cultivation at 25 °C for 7 days. After *B. cinerea* colonies grew to a diameter of approximately 5 cm, a 5 mm diameter sterile punch was used to cut a fungal plug from the edge of the plate, where the colonies were growing vigorously.

Six healthy one-year-old *A. corniculatum* saplings were randomly divided into two groups of three. All saplings were collected from Xiandao Park in Qinzhou, Guangxi, China (108°35′47.06″ E, 21°44′28.20″ N) and pre-cultured in the laboratory for 1 month under 12 h daylight at 25 ± 2 °C with 60–70% humidity, using a sterilized sand–soil substrate irrigated with 1/2 Hoagland solution.

Before inoculation, healthy and uniformly growing *A. corniculatum* leaves were selected, surface-disinfected with 75% ethanol, rinsed three times in sterile water, dried, and placed in a Petri dish lined with sterile wet gauze for moisture retention and storage. To minimize variation caused by wounding responses, both the control and treatment groups were subjected to a standardized wounding procedure prior to inoculation. A sterile dissecting needle was used to create a small wound at the inoculation site on each leaf. For the treatment group, a mycelial plug of *B. cinerea* was then attached to the wound site with sterile forceps, and a small amount of sterile water was added to enhance adhesion. For the control group, sterile PDA plugs without fungi were applied using the same procedure. All inoculated leaves were transferred to sealed humid containers and incubated under low-light conditions at 25 °C with near-saturated relative humidity. Lesion expansion was recorded every 24 h.

### 2.5. RNA Extraction, Library Preparation, Illumina Sequencing, and qPCR

For both control and treatment groups, three biologically independent one-year-old *A. corniculatum* saplings (from the six total saplings described in Section 2.4) were used as biological replicates. One fully expanded, healthy leaf was collected from each individual sapling at 72 h post-inoculation, flash-frozen in liquid nitrogen, and homogenized for total RNA extraction using TRIzol^®^ Reagent (Invitrogen, Carlsbad, CA, USA). To eliminate genomic DNA contamination, DNase I (Sigma-Aldrich, St. Louis, MO, USA) was added during RNA purification. RNA integrity was assessed using an Agilent 2100 Bioanalyzer (Agilent Technologies, Santa Clara, CA, USA), and only samples with an RNA Integrity Number ≥ 7 were used for downstream sequencing library preparation.

Sequencing libraries were constructed using a TruSeq Stranded mRNA LT Sample Prep Kit (Illumina, San Diego, CA, USA) following the manufacturer’s instructions. The libraries were then sequenced on an Illumina HiSeq™ 2500 platform, generating 150 bp paired-end reads. FastQc was used to conduct quality control on the original sequencing data. Fastp was employed to remove low-quality sequences and adapter contamination and obtain clean reads [29]. FeatureCounts (v2.1.1) was used for quantitative gene expression analysis, with gene expression levels expressed as fragments per million transcripts per kilobase of exon sequence (FPKM). The DESeq2 package was used to screen for differentially expressed genes, with the following screening criteria: |log_2_FoldChange| ≥ 1 and corrected *p*-value (padj/FDR) < 0.05 [30]. Under these criteria, expression changes in the treatment group compared to the control group were defined as significantly upregulated or significantly downregulated. The FPKM values were optimized by applying a log2(FPKM + 1) transformation, and an expression heatmap was generated for TLP family members in *A. corniculatum* using the pheatmap package in R 4.3.3.

The *Ac*TLP19-encoding gene exhibited a significant increase in transcript abundance in the RNA sequencing (RNA-seq) dataset. Therefore, this result was further verified by quantitative PCR (qPCR). RNA was extracted from the control and treatment groups using the method described above and reverse-transcribed into cDNA using Hifair III 1st Strand cDNA Synthesis SuperMix (YEASEN, Shanghai, China) according to the manufacturer’s instructions for subsequent qPCR analysis. Specific primers (Forward Primer: 5′- TGGACGGGTACAATGTTGGG-3′; Reverse Primer: 5′ CACGTGCTTGGTGTTGAGTG-3′) for *Ac*TLP19 were designed using Premier 6.0 software, and primer specificity was verified using 0.8% agarose gel electrophoresis. qPCR was performed on a qTower3 instrument (Analytik Jena AG, Jena, Germany) using SYBR Green fluorescent dye. The reaction mixture consisted of 5 μL 2× SYBR Green Pro Taq HS Premix (Accurate Biotechnology, Changsha, China), 0.5 μL each of forward and reverse primers (10 μM), 1 μL diluted cDNA template, and nuclease-free ddH_2_O added to a final volume of 10 μL. The PCR program was run as follows: initial incubation at 95 °C for 15 min, followed by 40 cycles of denaturation at 95 °C for 10 s, annealing at 59 °C for 15 s, and extension at 72 °C for 20 s. Melting curve analysis was performed immediately after amplification, with the temperature increasing from 60 °C to 95 °C, and fluorescence signals were collected at each 1 °C increment. The relative expression levels of target genes in each sample were calculated using the 2^−ΔΔCT^ method. Gene expression analysis was conducted with triplicate biological replicates, and expression levels in the control group were normalized to 1. Data processing and graph generation were performed using GraphPad Prism 9 software. Statistical significance among groups was first assessed using one-way ANOVA, followed by the least significant difference (LSD) post hoc test for pairwise comparisons. *p*-values were derived from the LSD test. β-Actin was used as the internal reference gene [6].

### 2.6. Subcellular Localization Prediction and Validation of Subcellular Localization of AcTLP19

Subcellular localization analysis for TLP was performed using the online site Plant-PLoc (http://www.csbio.sjtu.edu.cn/bioinf/plant/, accessed on 7 November 2025). The *Ac*TLP19 coding sequence was synthesized and cloned into the pGreenII 62-SK-GFP vector to generate an *Ac*TLP19-GFP fusion construct, which was confirmed by sequencing. The recombinant plasmid was transformed into the *Agrobacterium tumefaciens* strain GV3101 (pSoup-p19). A single colony was used to inoculate liquid Luria Bertani (LB) medium containing kanamycin (50 μg/mL) and rifampicin (25 μg/mL) and cultured at 28 °C with shaking at 220 rpm for 48 h. Bacterial cells were harvested by centrifugation at 7000 rpm for 2 min, washed once with 2-(N-morpholino)ethanesulfonic acid (MES) buffer (10 mM MES, 10 mM MgCl_2_, pH 5.6), and resuspended in the same buffer. The optical density at 600 nm (OD_600_) was adjusted to approximately 1.4. For co-localization assays, *A. tumefaciensstrain* cultures harboring *Ac*TLP19-GFP and the plasma membrane marker mCherry were mixed in equal volumes. Acetosyringone was added to a final concentration of 150 μM, and the mixture was incubated at 28 °C with shaking at 220 rpm for 1 h. The bacterial suspension was infiltrated into fully expanded leaves of 4–5-week-old *Nicotiana benthamiana* using a needleless syringe. The plants were maintained at 25 °C under a 16/8h light/dark photoperiod for 36 h. Fluorescence signals were observed using a laser scanning confocal microscope (ZEISS LSM 900, Jena, Thuringen, Germany). GFP fluorescence was excited at 488 nm and detected at 495–523 nm, and mCherry fluorescence was excited at 552 nm and detected at 580–650 nm. Leaf sections were treated with 1 M mannitol for approximately 10 min prior to observation for plasmolysis assays.

### 2.7. Expression and Purification of Recombinant AcTLP19 (rAcTLP19)

Based on previous research, the mature peptide of *Ac*TLP19 was codon-optimized for *Escherichia coli* and cloned into the pSmartI vector (containing His-SUMO tag) via BamHI/XhoI sites to generate pSmartI-*Ac*TLP19 (6228 bp, Supplementary Figure S1). The construct was transformed into *E. coli* BL21(DE3), and positive clones (kanamycin-resistant) were selected. Protein expression was induced with 0.5 mM isopropyl β-D-1-thiogalactopyranoside (IPTG) at 16 °C for 12 h. Cells were harvested and lysed using TieChui *E. coli* Lysis Buffer (ACE Biotechnology, Changzhou, China), and the His-SUMO-*Ac*TLP19 fusion protein in the supernatant was purified via Ni-column affinity chromatography. The His-SUMO tag was cleaved using a SUMO protease, yielding tag-free recombinant *Ac*TLP19 (r*Ac*TLP19), which was confirmed by sodium dodecyl sulfate–polyacrylamide gel electrophoresis (SDS-PAGE). The protein concentration was determined using a BSA Protein Assay Kit (Beyotime, Shanghai, China) following the manufacturer’s instructions, and aliquots were stored at −80 °C. The freeze-dried polypeptide powder was sent to Wininnovate Bio Company (Shenzhen, Guangdong, China) for qualitative identification using liquid chromatography–mass spectrometry (LC-MS).

### 2.8. Antimicrobial Activity of rAcTLP19

Antifungal activity against *B. cinerea* and *F. oxysporum* was assessed by determining the half-maximal inhibitory concentration (MIC_50_) using a modified broth microdilution assay, following the Clinical and Laboratory Standards Institute (CLSI) M38 guidelines. Conidial suspensions were harvested from 7-day-old cultures grown on PDA and standardized to a final density of 1 × 10^4^ CFU/mL in RPMI-1640 medium buffered with 3-(N-morpholino)propanesulfonic acid at pH 7.0. Two-fold serial dilutions of the test compounds were prepared in 96-well microtiter plates, and an equal volume of the standardized conidial suspension was added to each well. Plates were incubated statically at 25 °C for 48 h, after which fungal growth was quantified by measuring the OD_595_. The MIC_50_ was defined as the lowest compound concentration that reduced OD_595_ by ≥50% compared to the solvent-only growth control. Appropriate solvent controls (e.g., ≤1% *v*/*v* DMSO) were included to exclude nonspecific effects, and all experiments were conducted in triplicate, with three biologically independent replicates. Amphotericin B was used as the positive control.

### 2.9. Statistical Analysis

GraphPad Prism 9.0 (GraphPad, San Diego, CA, USA) was used for statistical analysis. For comparisons involving more than two groups, one-way ANOVA was performed, followed by the least significant difference (LSD) test for pairwise comparisons when ANOVA indicated significant differences (*p* < 0.05). All data are presented as the mean ± standard error (SE). A *p*-value < 0.05 was considered statistically significant.

## 3. Results

### 3.1. Identification of TLP Genes in A. corniculatum and Chromosomal Location

**Figure 1:**
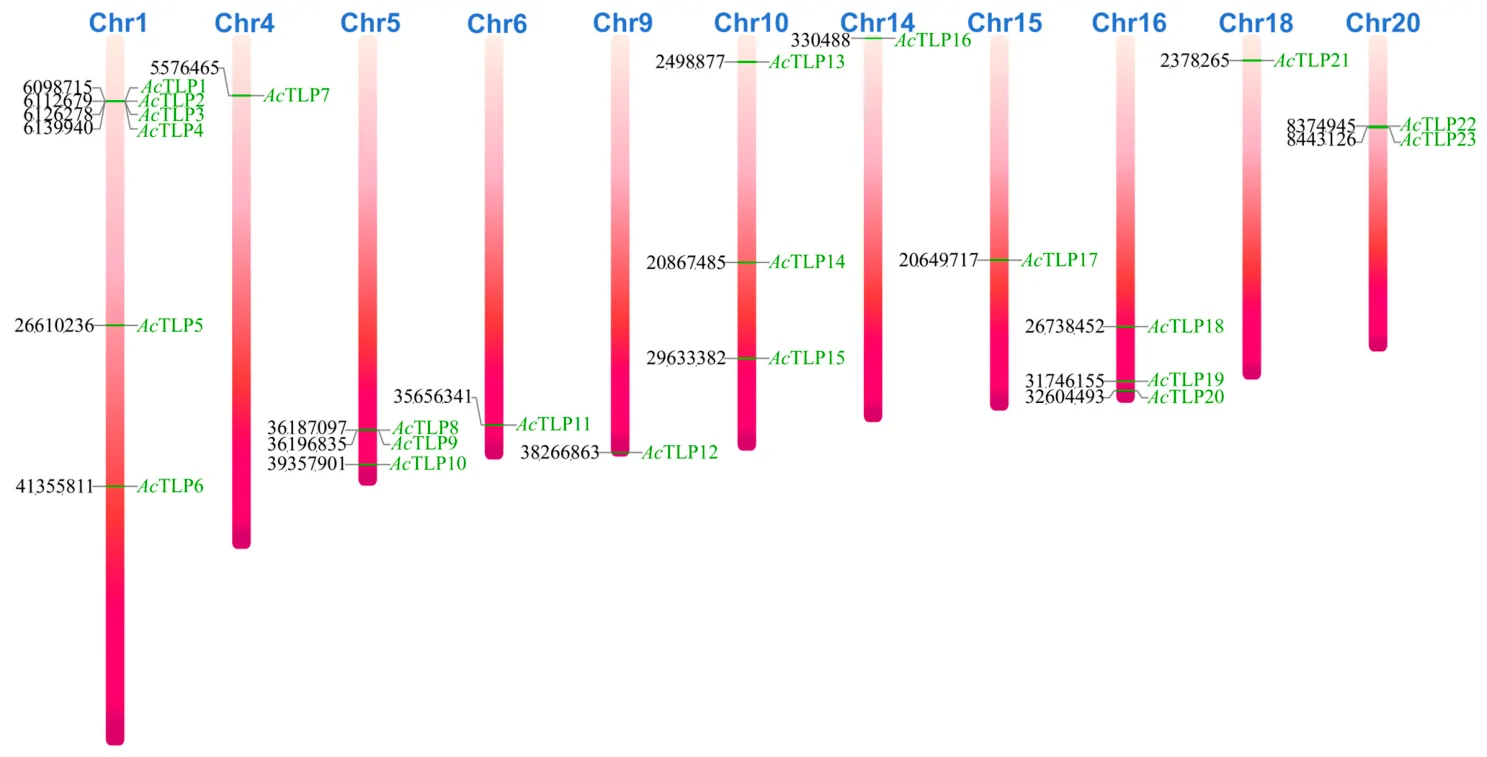
Chromosomal locations of TLP family members in *A. corniculatum*.

This analysis revealed 23 putative TLP family members in the *A. corniculatum* genome. These candidate genes were systematically named based on their sequential positions on the chromosomes. As shown in Figure 1, the 23 identified TLP family members were distributed across 11 chromosomes in the *A. corniculatum* genome. Chromosome 1 had the most candidate TLP family genes (6).

### 3.2. Physicochemical Properties Prediction

ProtParam analysis revealed significant variation in molecular weights among TLP family members in *A. corniculatum* (Table 1). *Ac*TLP8 was the smallest member with only 140 amino acids, while *Ac*TLP16 was the largest member with 1056 amino acids. Among the identified members, AcTLP10 (1025 aa) and AcTLP16 (1056 aa) exceeded the typical TLP length range of 200–300 amino acids. Domain architecture analysis revealed that the extended N-terminal region of *Ac*TLP10 consisted of a legume lectin domain and a serine/threonine kinase catalytic domain and that the N-terminus of *Ac*TLP16 harbored a dsDNA-specific endonuclease domain (Supplementary Table S1 and Figure S2). In both cases, the C-terminal region encoded a complete thaumatin domain (PF00314), confirming their classification as TLP family members despite their unusual multi-domain organization.

As shown in Table 1, *Ac*TLP9 had the highest isoelectric point (pI), whereas *Ac*TLP21 had the lowest. *Ac*TLP6, *Ac*TLP7, *Ac*TLP10, *Ac*TLP11, *Ac*TLP13, *Ac*TLP14, *Ac*TLP18, *Ac*TLP19, *Ac*TLP20, *Ac*TLP21, *Ac*TLP22, and *Ac*TLP23 possessed the predicted signal peptides, while the remaining members lacked this feature. The instability index of most TLP family members was above 40, indicating that they are classified as unstable proteins according to the ExPASy ProtParam criteria. In particular, *Ac*TLP13 demonstrated extremely high stability. However, *Ac*TLP6 exhibited the highest instability index (57.46), followed by *Ac*TLP15 (55.66). The aliphatic index of TLP family members in *A. corniculatum* was generally high, suggesting that these proteins had good thermal stability. The overall average GRAVY value of TLP family members in *A. corniculatum* was close to zero or negative, indicating that most were hydrophilic proteins, which may be related to their functions in aqueous environments, such as extracellular or vacuole environments.

### 3.3. Phylogenetic and Collinearity Analysis of TLP Family Members of A. corniculatum

**Figure 2:**
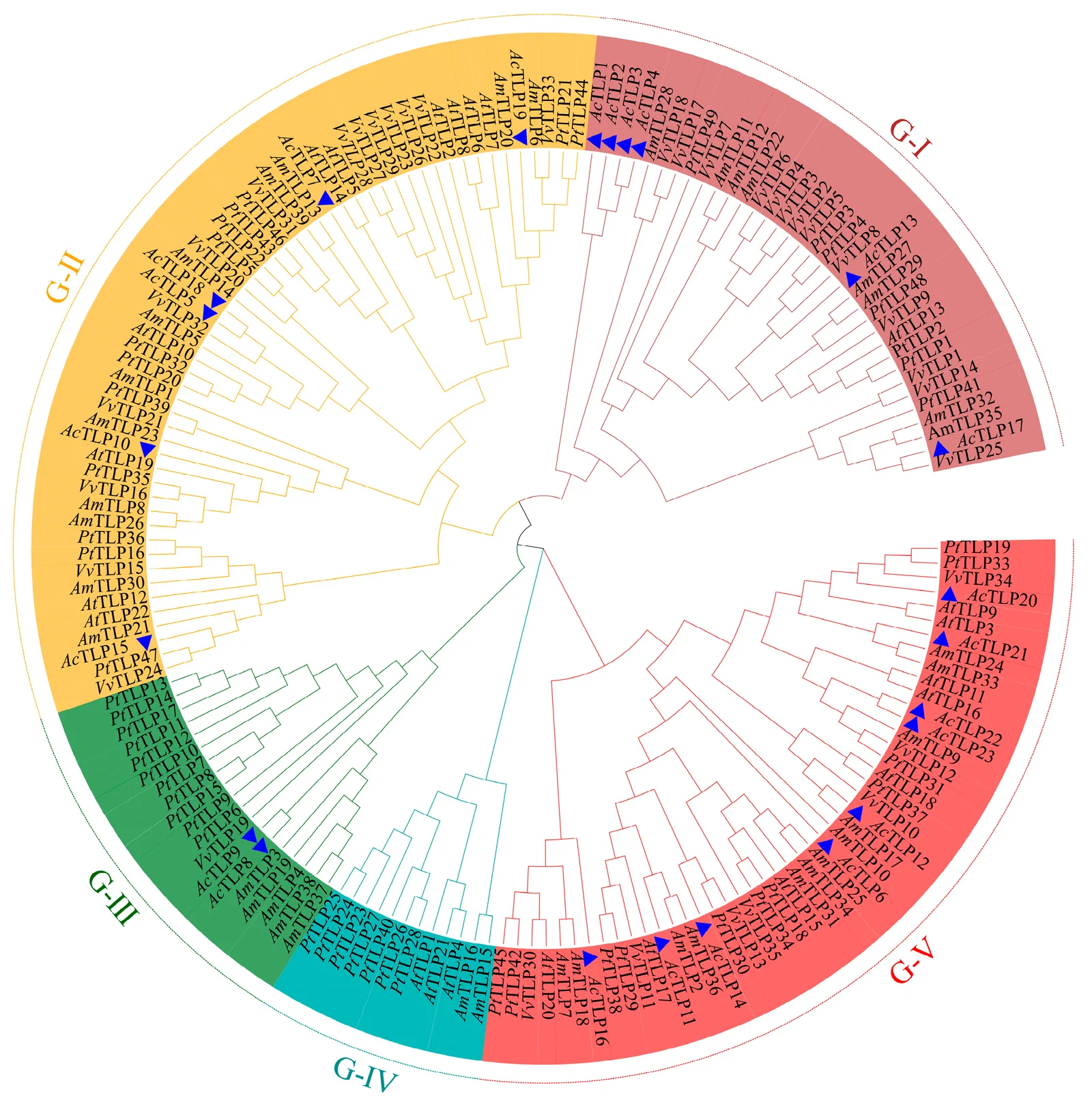
Phylogenetic analysis of TLP family members from *A. corniculatum*, *A. marina*, *V. vinifera*, *A. thaliana*, and *P. trichocarpa*. TLP family members of *A. corniculatum* are denoted by a blue triangle.

All TLP family members of *A. corniculatum*, *A. marina*, *V. vinifera*, *A. thaliana*, and *P. trichocarpa* were divided into five groups (Groups I–V). Among them, Group V had the most members, including *Ac*TLP6, *Ac*TLP11, *Ac*TLP13, *Ac*TLP14, *Ac*TLP20, *Ac*TLP22, and *Ac*TLP23. Groups I, II, and III had six, seven, and two TLP family members in *A. corniculatum*, respectively, and Group IV had no TLP family members in *A. corniculatum* (Figure 2).

**Figure 3:**
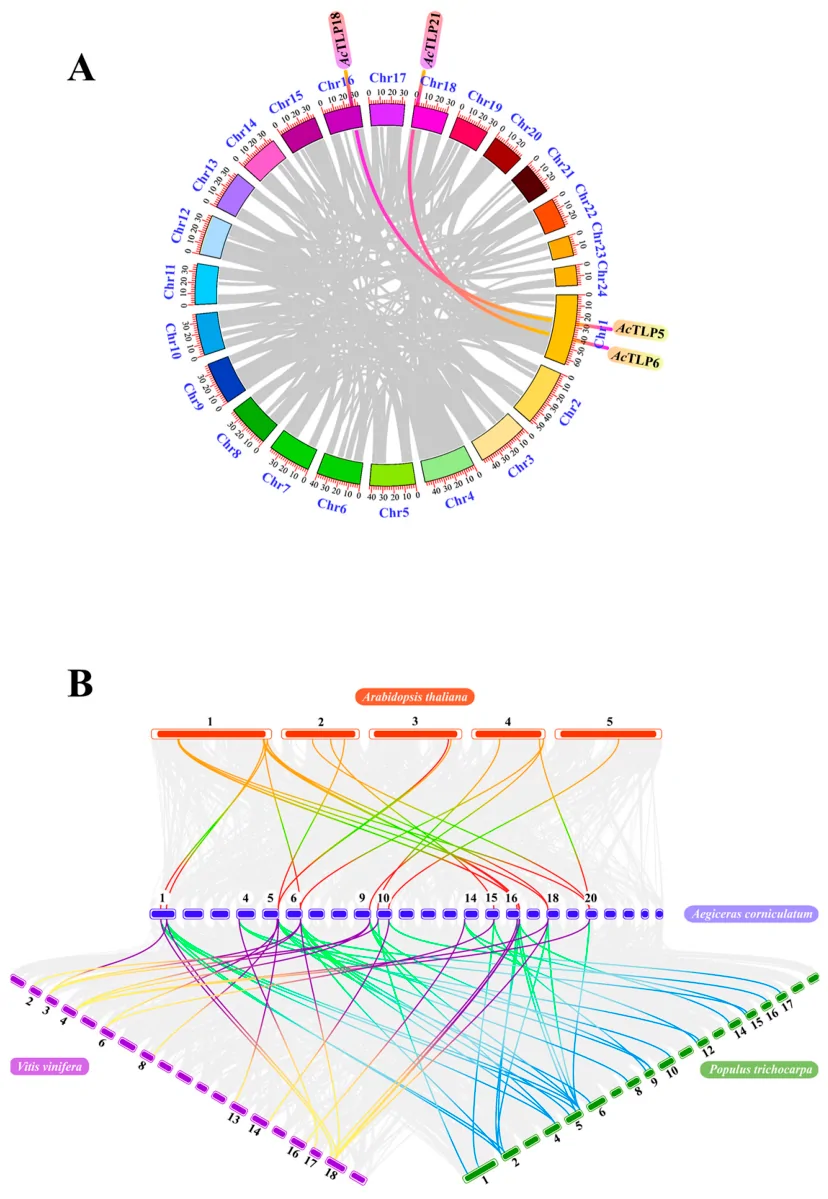
Analysis of evolutionary relationships among TLP family members. (**A**) Fragment duplication events in the *A. corniculatum* genome. (**B**) Synteny analysis of TLP family members among *A. corniculatum*, *V. vinifera*, *P. trichocarpa*, and *A. thaliana*.

The evolution of the TLP family in *A. corniculatum* involved two segmental duplication events, namely the duplication of *AcTLP5/AcTLP18* and *AcTLP6/AcTLP21* (Figure 3A). The non-synonymous substitution rate (Ka), synonymous substitution rate (Ks), and their ratio (Ka/Ks) for each duplicated gene pair are shown in Table 2. The Ka/Ks ratios varied considerably among the seven tandem duplication pairs identified on chromosomes 1 and 20. The Ka/Ks ratio for the *AcTLP3/AcTLP4* pair could not be calculated (denoted as “NA”), likely due to their nearly identical coding sequences, resulting in zero synonymous substitutions. *AcTLP2/AcTLP3*, *AcTLP2/AcTLP4*, and *AcTLP23/AcTLP22* exhibited Ka/Ks ratios greater than 1 (1.01, 1.01, and 1.75, respectively), suggesting positive selection. However, given the limited number of tandem duplication events available for analysis (*n* = 3 pairs with Ka/Ks > 1), this observation should be interpreted with caution and does not constitute conclusive evidence of widespread positive selection in the TLP family. The Ka/Ks ratios of the remaining gene pairs were all less than 1, indicating that they were predominantly under purifying (negative) selection pressure during evolution.

Divergence times for duplicated gene pairs were estimated using the formula T = Ks/(2λ), where λ represents the neutral synonymous substitution rate. Based on the previously established rate for dicotyledonous plants, λ was set to 1.5 × 10^−8^ substitutions per synonymous site per year [31]. This calculation yielded divergence times ranging from approximately 0.33 MYA (*Ac*TLP22/*Ac*TLP23) to 42.30 MYA (*Ac*TLP6/*Ac*TLP21), as detailed in Table 2. These estimates assume a constant molecular clock and may be influenced by saturation effects for older duplication events with Ks values approaching 2.5.

Figure 3B and Supplementary Table S2 show the results of collinearity analysis of TLP family members among different species. Seventeen collinear gene pairs of TLP family members were identified between *A. corniculatum* and *A. thaliana*. Between *A. corniculatum* and *P. trichocarpa* and between *A. corniculatum* and *V. vinifera*, there were 18 and 27 collinear gene pairs of TLP family members, respectively.

### 3.4. Genetic Structure and Motif Identification of TLP Family Members of A. corniculatum

**Figure 4:**
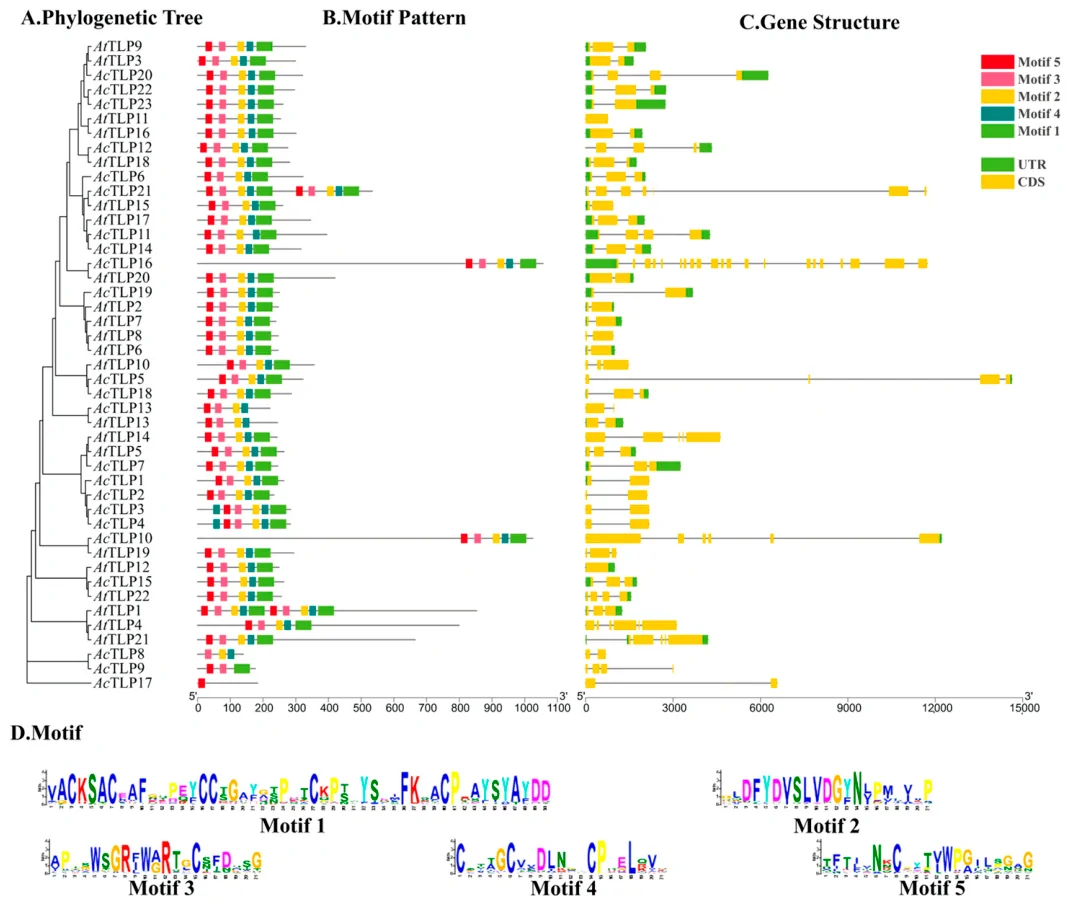
Gene structure and motif analysis of TLP family members in *A. corniculatum*. (**A**) Unrooted phylogenetic tree constructed based on TLP family members in *A. corniculatum*. (**B**) Conserved motif distribution of TLP family members in *A. corniculatum*. (**C**) Exon–intron composition analysis. The black lines denote intron positions. (**D**) Details on conserved motifs.

As shown in Figure 4, the gene structures of *Ac*TLP2, *Ac*TLP3, *Ac*TLP4, *Ac*TLP8, *Ac*TLP9, *Ac*TLP13, and *Ac*TLP17 do not contain untranslated regions. The *Ac*TLP16 gene structure had the most introns and exons, with 21 exons and 20 introns.

Except for *Ac*TLP21, most TLP family members were composed of only one set of motifs (1, 2, 3, 4, and 5). Compared to other members of the TLP family, *Ac*TLP3 and *Ac*TLP4 had an additional motif 4.

### 3.5. Upstream Gene Element Analysis

**Figure 5:**
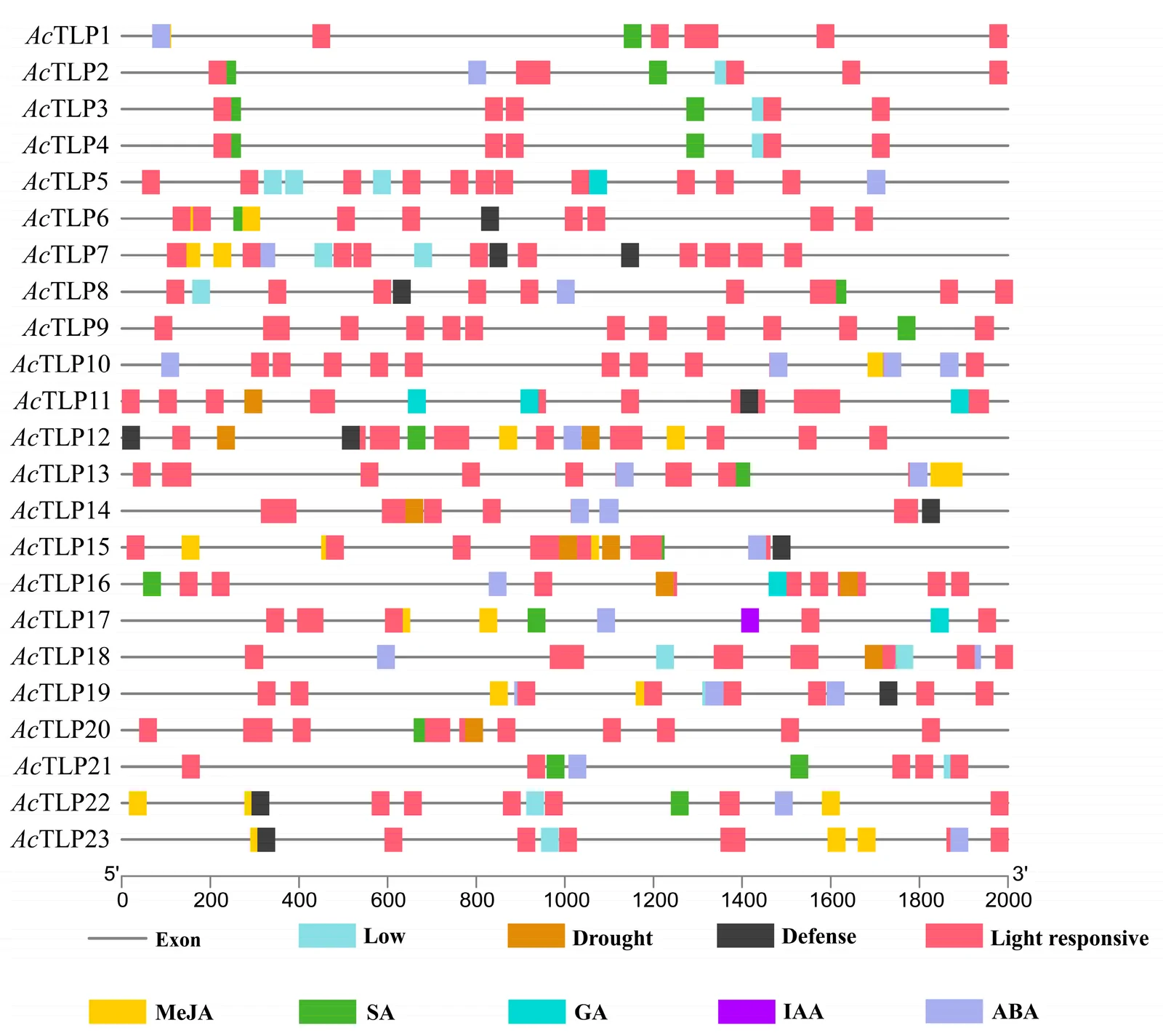
Upstream regulatory element analysis of TLP family members in *A. corniculatum*.

The region upstream of *Ac*TLP17 had an auxin (IAA) element. The regions upstream of *Ac*TLP6, *Ac*TLP7, *Ac*TLP10, *Ac*TLP12, *Ac*TLP13, *Ac*TLP15, *Ac*TLP17, *Ac*TLP18, *Ac*TLP19, *Ac*TLP22, and *Ac*TLP23 had methyl jasmonate (MeJA) components. The regions upstream of *Ac*TLP1, *Ac*TLP2, *Ac*TLP3, *Ac*TLP4, *Ac*TLP6, *Ac*TLP8, *Ac*TLP9, *Ac*TLP12, *Ac*TLP15, *Ac*TLP16, *Ac*TLP17, *Ac*TLP20, *Ac*TLP21, and *Ac*TLP22 had salicylic acid (SA) elements. Abscisic acid responsive elements (ABREs) were identified in the promoters of most TLP family members, except *Ac*TLP3, *Ac*TLP4, *Ac*TLP6, *Ac*TLP9, *Ac*TLP11, *Ac*TLP16, *Ac*TLP17, and *Ac*TLP20. *Ac*TLP18 had four ABREs. The regions upstream of *Ac*TLP6, *Ac*TLP7, *Ac*TLP8, *Ac*TLP11, *Ac*TLP12, *Ac*TLP14, *Ac*TLP15, *Ac*TLP19, *Ac*TLP22, and *Ac*TLP23 had regulating elements capable of defense responses. Both *Ac*TLP7 and *Ac*TLP12 had two regulating elements that activate defenses. The regions upstream of *Ac*TLP11, *Ac*TLP12, *Ac*TLP14, *Ac*TLP15, *Ac*TLP16, *Ac*TLP18, and *Ac*TLP20 had regulatory elements that respond to drought stress (Figure 5).

### 3.6. Responses to Pathogenic Microbial Threats

**Figure 6:**
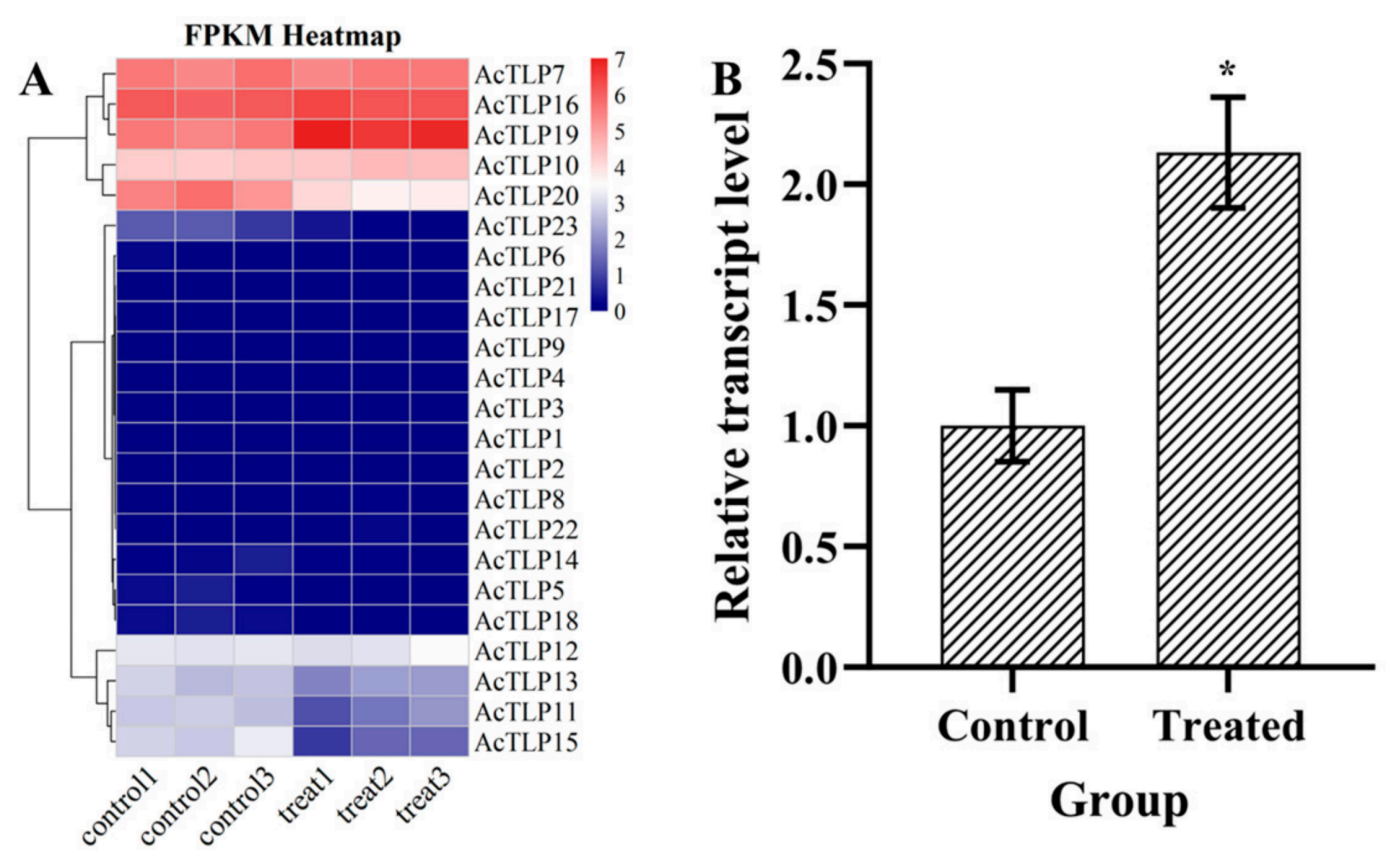
Expression analysis of TLP family members in *A. corniculatum* leaves in response to *B. cinerea* infection in 72 h. (**A**) Expression heatmap of TLP family members in *A. corniculatum*. (**B**) Validation of *AcTLP19* mRNA levels using qPCR. * *p*-value < 0.05.

The expression profiles of TLP family members in *A. corniculatum* leaves infected with *B. cinerea* were systematically analyzed to elucidate their functional roles in plant immune responses. As shown in Figure 6A, infection significantly upregulated *Ac*TLP19 mRNA levels, but the mRNA levels of *Ac*TLP5, *Ac*TLP11, *Ac*TLP14, *Ac*TLP15, *Ac*TLP20, *Ac*TLP22, and *Ac*TLP23 were significantly downregulated. No statistically significant changes were observed in the transcript abundance of other TLP-encoding genes in *A. corniculatum*. qPCR validation confirmed the robust and specific upregulation of *Ac*TLP19 following *B. cinerea* infection (Figure 6B).

### 3.7. Subcellular Localization

**Figure 7:**
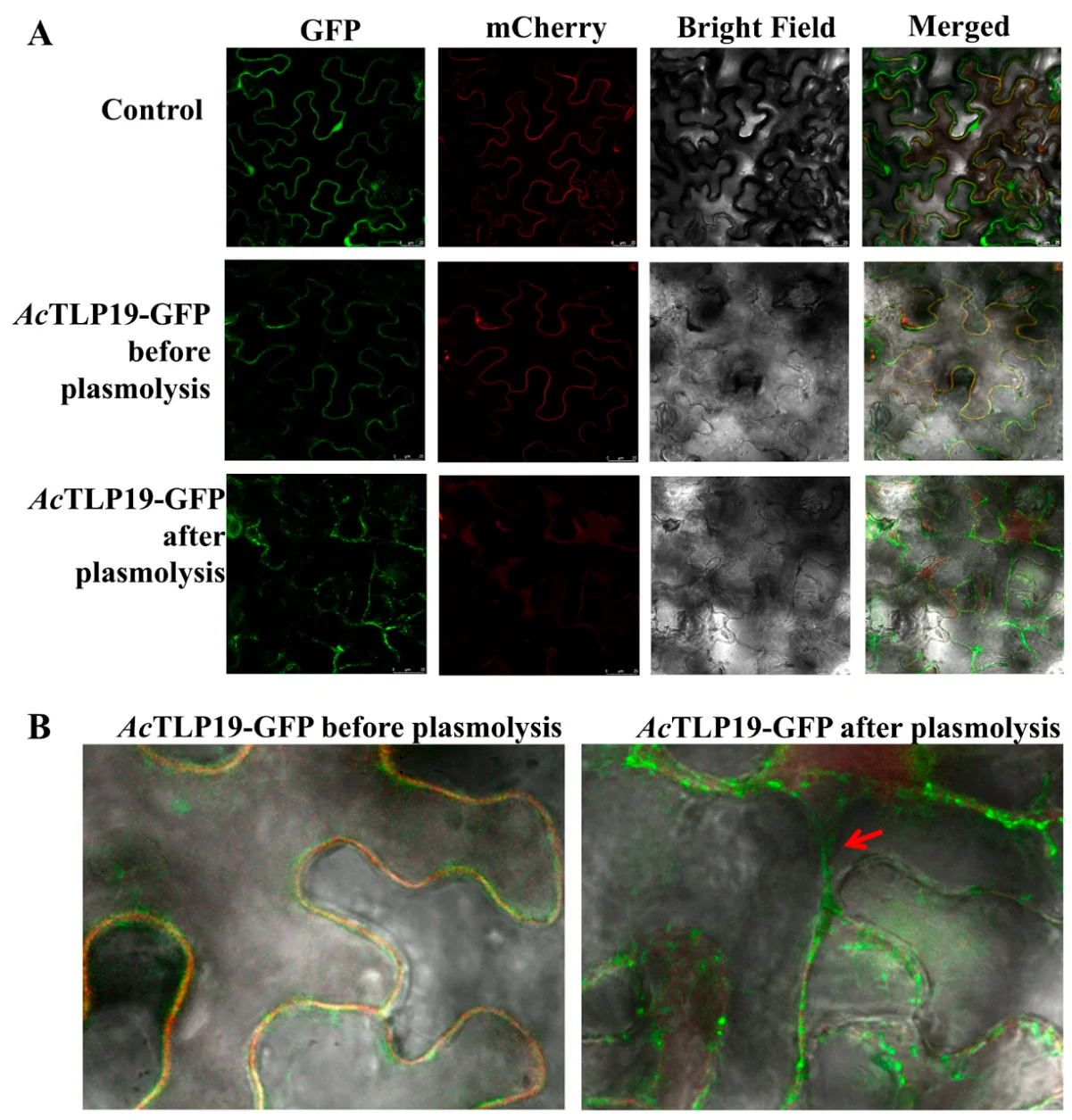
Subcellular localization of *Ac*TLP19. (**A**) Comparative confocal microscopy imaging between the control group (expressing only free GFP and mCherry markers) and the experimental group (expressing AcTLP19-GFP) before and after plasmolysis. (**B**) Local magnification images of the experimental group (AcTLP19-GFP) before and after plasmolysis. The red arrow indicates the green fluorescence signal at the edge of the contracted protoplast.

According to the Plant-PLoc prediction results, the subcellular localization of TLP family members was diverse, mainly concentrated in chloroplasts and extracellular regions (Table 3). Members located in chloroplasts included *Ac*TLP1, *Ac*TLP2, *Ac*TLP3, *Ac*TLP4, *Ac*TLP5, *Ac*TLP15, *Ac*TLP16, and *Ac*TLP20; extracellular members included *Ac*TLP6, *Ac*TLP11, *Ac*TLP12, *Ac*TLP14, *Ac*TLP18, *Ac*TLP19, *Ac*TLP21, *Ac*TLP22, and *Ac*TLP23. The remaining members were located in the cell wall, vacuoles, mitochondria, and cytoplasm. Experimental verification showed that the *Ac*TLP19 protein co-localized with cell membrane markers (Figure 7A). The plasmolysis experiment indicated that some of the *Ac*TLP19 protein was secreted outside the cell (Figure 7B).

### 3.8. Heterologous Expression of AcTLP19

**Figure 8:**
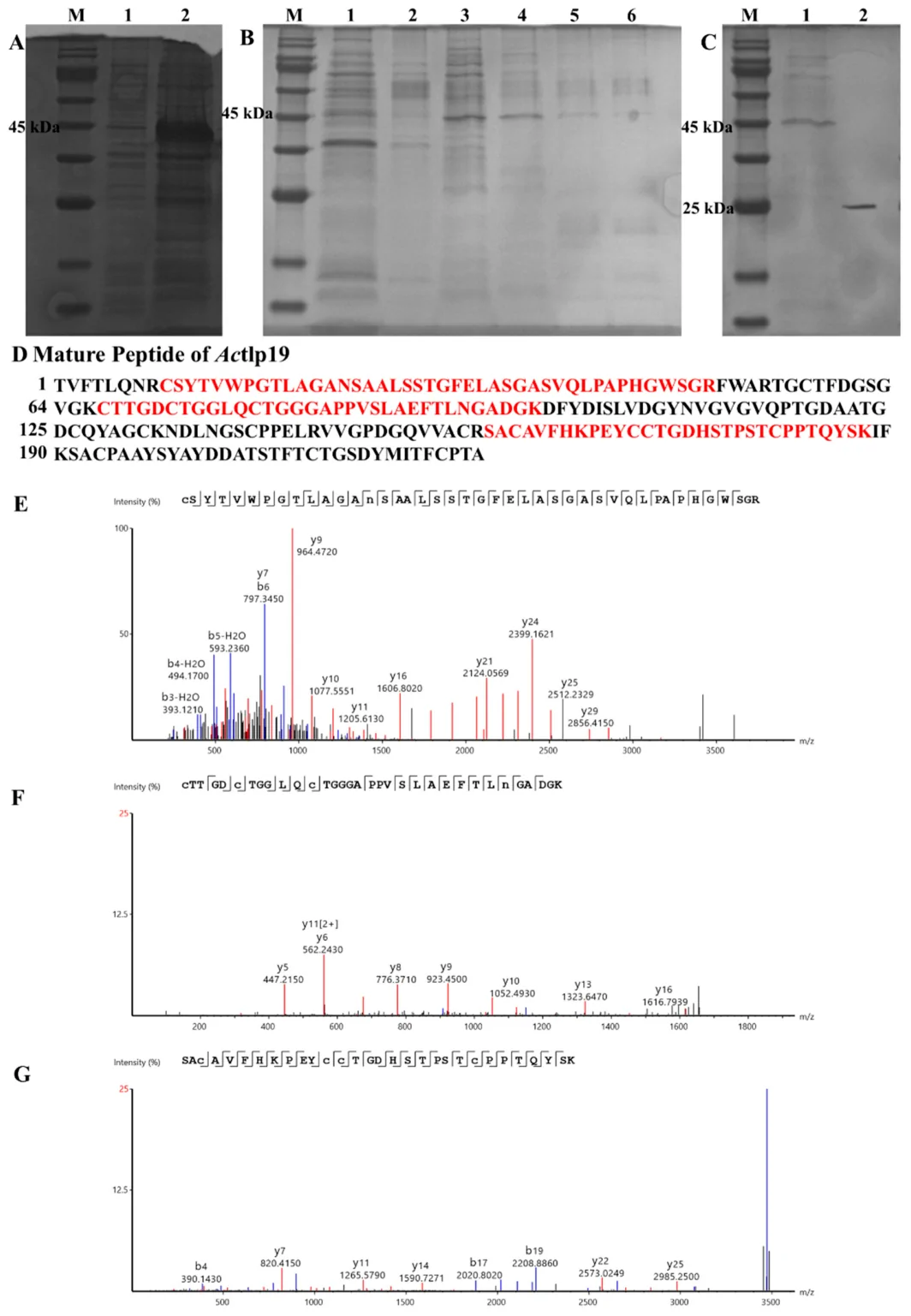
Acquisition process and MS spectrum analysis of r*Ac*TLP19. (**A**) SDS-PAGE analysis of recombinant r*Ac*TLP19 expressed with a SUMO tag in *E. coli*. Lane M, protein marker; lane 1, total protein obtained from *E. coli* without induction; lane 2, total protein obtained from *E. coli* with IPTG induction. (**B**) His-SUMO-*Ac*TLP19 purified with nickel column chromatography. Lane M, protein marker; lane 1, protein not caught by the nickel column; lane 2, equilibration buffer; lane 3, eluent with 50 mM imidazole; lane 4, eluent with 100 mM imidazole; lane 5, eluent with 200 mM imidazole; lane 6, eluent with 500 mM imidazole. (**C**) SDS-PAGE of r*Ac*TLP19 without the SUMO tag. Lane M, protein marker; lane 1, His-SUMO-*Ac*TLP19 before treatment with the SUMO enzyme; lane 2, r*Ac*TLP19 obtained after removing the SUMO tag. (**D**) Alignment of mass spectrometry results with the r*Ac*tlp19 sequence. The amino acids highlighted in red were detected by LC-MS, while the amino acids marked in black were not detected by LC-MS. (**E**) MS spectrum of “CSYTVWPGTLAGANSAALSSTGFELASGASVQLPAPHGWSG.” (**F**) MS spectrum of “CTTGDCTGGLQCTGGGAPPVSLAEFTLNGADGK.” (**G**) MS spectrum of “SACAVFHKPEYCCTGDHSTPSTCPPTQYSK.” The red, blue, and black lines are the y ions, b ions, and noise signals detected by mass spectrometry, respectively.

Using pSmartI as the vector template, the mature peptide of *Ac*TLP19 was fused to the C-terminus of the His-SUMO tag. SDS-PAGE (Figure 8A) revealed differences in the band patterns of 45 kDa proteins expressed by the bacteria before and after IPTG induction. This band corresponded to the expected size of the His-SUMO-*Ac*TLP19 fusion protein, which consisted of a His-SUMO tag (approximately 18 kDa) and a mature *Ac*TLP19 peptide (23.02 kDa). His-SUMO-*Ac*TLP19 was eluted from the Ni-column using gradient elution imidazole eluent, with an optimal elution concentration of 500 μM (Figure 8B, lane 6). After treatment with the SUMO enzyme, recombinant *Ac*TLP19 (r*Ac*TLP19) without the His-SUMO tag was obtained and characterized on SDS-PAGE as approximately 25 kDa (Figure 8C). The r*Ac*TLP19 sequence was analyzed using Mascot Distiller (v2.7). Three peptides were identified with an amino acid sequence coverage of 47.08% (Figure 8D–G).

### 3.9. Antifungal Activity of Recombinant AcTLP19

The results of in vitro antibacterial activity assays indicated that r*Ac*TLP19, even at a high concentration of 128 μg/mL, did not show significant inhibitory effects on *B. cinerea* or *F. oxysporum* (Table 4).

## 4. Discussion

TLPs are a class of plant pathogenesis-related proteins that participate in biotic stress responses and disease resistance defense processes [15,16,32]. They play critical roles in molecular mechanisms, including mediating antifungal activity regulation, enhancing plant stress tolerance, responding to environmental stressors, and contributing to the coordinated defense responses of plants against both biotic and abiotic stressors [22,32,33]. However, research on TLP family members in mangrove species remains limited. A total of 23 TLP family members were identified in *A. corniculatum*. This number was lower than that in *A. thaliana* (28), *O. sativa* (30), and *Cucumis melo* (28) [22,34]. This observation may reflect the genomic streamlining strategy adopted by this species in the unique mangrove habitat, where it copes with biotic stress by retaining multifunctional core genes rather than undergoing large-scale family expansion, which is also associated with species-specific defense requirements [35,36,37].

TLP family members typically consist of 200–300 amino acids [38]. For example, the amino acid lengths of TLP family members in *A. thaliana* range from 241 to 863 [22,38]. In *A. corniculatum*, however, *Ac*TLP10 and *Ac*TLP16 both exceeded 1000 amino acids due to their long N-terminal extension regions (Figure 4B). Further analysis of the conserved domains showed that the N-terminus of *Ac*TLP10 consisted of a legume lectin domain and a serine/threonine kinase catalytic domain, and its C-terminus is a typical TLP domain. In contrast, the N-terminus of AcTLP16 was identified to have a dsDNA-specific endonuclease domain. Such multi-domain fusion events in plants are usually closely related to developmental regulation, stress responses, and immune responses [39,40]. Given that mangrove plants have long been exposed to extreme environments, such as high salinity and drought [41], it is tempting to speculate that strong selective pressure may have contributed to the evolution of AcTLP10 and AcTLP16 into multifunctional proteins through domain fusion. However, this hypothesis was not directly tested in the present study. Future experiments, such as comparative genomics across mangrove species and functional assays of the fused domains, are required to validate whether these domain architectures indeed confer adaptive advantages under stress conditions.

The 23 TLP family members in *A. corniculatum* were unevenly distributed across 11 chromosomes, a pattern also observed in *A. thaliana*, *O. sativa*, and *V. vinifera*, indicating that this non-uniform distribution is a common feature of plant TLP family members and may be associated with the chromosome structure, gene duplication events, and functional selective pressure [17,22]. This study revealed that tandem duplications among TLP family members in *A. corniculatum* occurred exclusively on chromosomes 1 and 20, with the frequency and scale both significantly lower than those in *V. vinifera* and *C. melo* [17,34]. This observation reflects substantial differences in the strategies for gene family expansion among species. In *V. vinifera* and *C. melo*, the expansion of TLP family members primarily relies on local chromosomal tandem duplications, forming dense gene clusters to meet demands related to complex pathogen interactions and fruit development [17,34]. As a mangrove species, *A. corniculatum* has recently undergone whole-genome duplication, suggesting that the expansion of its TLP family members depends on the retention and functional diversification of duplicated genes following whole-genome duplication [26,42]. The limited number of tandem duplications in *A. corniculatum* could be attributed to several factors, including whole-genome duplication followed by differential gene retention, or purifying selection against unstable tandem-repeat regions in the extreme mangrove habitat. However, the specific mechanisms underlying this pattern remain to be directly investigated. Population-level genomic analyses will be necessary to determine whether active elimination or neutral evolutionary processes shaped the current TLP family architecture [42,43,44].

Phylogenetic analysis showed that *Vv*TLP1 and *Ac*TLP17 had a relatively close genetic relationship on the evolutionary tree. Studies have shown that *Vv*TLP1 significantly inhibits the in vitro spore germination and mycelial growth of *B. cinerea* [18]. It is tempting to speculate that *Ac*TLP17 may similarly contribute to pathogen defense in *A. corniculatum*. However, this hypothesis requires experimental validation through heterologous expression or gene knockout assays. *Vv*TLP29 has been reported to be involved in disease resistance responses [17]. *Vv*TLP29 and *Ac*TLP19 are highly clustered, and transcriptome data showed that both significantly upregulate their expression after fungal infection. Therefore, we speculated that *Ac*TLP19 is also likely to function in pathogen infection resistance, and further functional verification is necessary. Furthermore, *Ac*TLP8 and *Ac*TLP9, along with the known *Pt*TLP6, which is involved in flower development regulation, belong to the third group in the phylogenetic tree. *Pt*TLP6 overexpression has been shown to result in an early flowering phenotype [23]. Based on this phylogenetic clustering, we predict that *Ac*TLP8 and *Ac*TLP9 may be involved *in* the growth and development of *A. corniculatum*. However, this inference is based solely on evolutionary relatedness and requires experimental validation through expression analysis and functional assays.

After plants are stressed by pathogenic microorganisms, defense-related transcription factors bind to the cis-acting elements of target genes, activating gene expression that confers resistance against pathogen invasion [45,46,47]. As a plant hormone and signaling molecule related to damage, MeJA can stimulate the expression of plant defense genes and induce plant chemical defense [48,49]. The promoter regions of *Ac*TLP6, *Ac*TLP7, *Ac*TLP10, *Ac*TLP12, *Ac*TLP13, *Ac*TLP15, *Ac*TLP17, *Ac*TLP18, *Ac*TLP19, *Ac*TLP22, and *Ac*TLP23 contain cis-acting elements responsive to MeJA. In our case, only *Ac*TLP19 was upregulated, reflecting its higher sensitivity to the endogenous defense signals elicited by the pathogen or more favorable promoter architectures for fungal-induced activation, as similarly reported for TLP family members in garlic and banana under fungal infection [19,20].

Natural antimicrobial peptides commonly contain signal peptides and can be specifically transported to the extracellular matrix via the classical secretory pathway [50]. Prediction analysis indicates that TLP family members in *A. corniculatum* containing signal peptides include *Ac*TLP6, *Ac*TLP7, *Ac*TLP10, *Ac*TLP11, *Ac*TLP13, *Ac*TLP14, *Ac*TLP18, *Ac*TLP19, *Ac*TLP20, *Ac*TLP21, *Ac*TLP22, and *Ac*TLP23. Subcellular localization predictions suggest that, except for *Ac*TLP10, all other TLP family members with signal peptides in *A. corniculatum* are either located or are likely to be present in the extracellular matrix. This extracellular localization is consistent with a potential role in defense responses, as many secreted PR proteins function in the apoplast to counteract invading pathogens [50,51].

The expression patterns of TLP family members exhibit pathogen specificity and temporal dynamics. For instance, the expression responses of TLP family members in grapes vary under infections by different pathogens. In *V. vinifera*, 23 TLP family members are upregulated by anthracnose, 14 by powdery mildew, and 19 by *B. cinerea*; among them, *Vv*TLP3, 6, 7, 8, 12, and 29 are all significantly upregulated after infection by the three pathogens [17]. Moreover, the expression of TLP family members is time-specific. For example, after *R. cerealis* infects wheat H83, *Ta*TLP46 is significantly upregulated at 36 h and returns to the control level at 72 h [21]. In this study, we only measured the response of *A. corniculatum* to *B. cinerea* infection after 72 h. Therefore, genes with no significant differences and that were downregulated may still have an anti-pathogen function.

The genes involved in plant pathogen defense are usually upregulated after microbial infection. *Ac*TLP19 was significantly upregulated 72 h after *B. cinerea* infection. The extracellular localization of *Ac*TLP19, as demonstrated by GFP fluorescence and plasmolysis assays, aligns with the canonical behavior of PR proteins, which are typically secreted to the apoplast. While this localization is consistent with a potential defensive role, it does not constitute direct evidence of antimicrobial activity, particularly given the absence of detectable antifungal activity against *B. cinerea* and *F. oxysporum* in our recombinant *Ac*TLP19 assays. The reducing environment of the *E. coli* cytoplasm may prevent proper formation of the eight conserved disulfide bonds characteristic of PR-5 proteins, potentially leading to misfolding and loss of activity [52,53]. Eukaryotic expression systems may be required to obtain correctly folded proteins. Furthermore, the in vitro assay conditions (RPMI-1640, pH 7.0) differ from the acidic apoplastic environment (pH ~5.0–6.0), where *Ac*TLP19 naturally accumulates [54]. pH and ionic conditions influence antifungal peptide activity [55]. Therefore, the absence of detectable direct antifungal activity does not rule out a defensive role for *Ac*TLP19, and it may participate in immunity through indirect mechanisms, such as recognizing pathogen molecules to trigger downstream signaling, inhibiting pathogen-secreted virulence factors, and reinforcing the cell wall structure [51,56]. Future studies employing gene silencing or knockout approaches in *A. corniculatum*, combined with protein production in eukaryotic systems, are necessary to clarify its precise mode of action.

## 5. Conclusions

In this study, 23 TLP family members were identified in *A. corniculatum*, and their phylogenetic relationships, gene structures, promoter elements, and expression patterns were comprehensively analyzed. *Ac*TLP19 was significantly upregulated in response to *B. cinerea* infection and localized to the extracellular space. Although recombinant *Ac*TLP19 showed no direct antifungal activity in vitro, its pathogen-induced expression suggested a role in mangrove defense. However, whether *Ac*TLP19 functions via indirect immune mechanisms, requires eukaryotic post-translational modifications for activity, or acts through other yet-to-be-determined pathways remains to be directly tested in future studies. These findings enhance our understanding of the molecular basis of disease resistance in mangroves and provide a foundation for future conservation strategies.

## Figures and Tables

**Table 1:** Physicochemical property prediction of TLP family members in *A. corniculatum*.

Name	Size (Number of Amino Acids)	pI	Cys Number	Arg + Lys Number	Instability Index	Aliphatic Index	GRAVY	Signal Peptide
*Ac*TLP1	263	8.27	17	28	54.13	62.36	−0.278	NO
*Ac*TLP2	233	8.81	15	27	45.39	59.91	−0.024	NO
*Ac*TLP3	284	9.14	14	35	46.50	75.25	−0.243	NO
*Ac*TLP4	284	9.14	14	35	46.50	75.28	−0.243	NO
*Ac*TLP5	322	8.35	19	34	31.36	81.46	−0.025	NO
*Ac*TLP6	322	4.92	17	14	57.46	66.96	0.008	YES
*Ac*TLP7	245	8.97	17	23	41.26	72.57	0.001	YES
*Ac*TLP8	140	4.98	8	11	40.54	88.36	−0.064	NO
*Ac*TLP9	176	9.21	11	16	44.03	64.26	−0.044	NO
*Ac*TLP10	1025	8.67	23	106	39.95	84.33	−0.179	YES
*Ac*TLP11	395	4.58	19	17	39.44	52.66	−0.040	YES
*Ac*TLP12	275	8.11	16	23	38.33	67.45	−0.076	NO
*Ac*TLP13	221	4.85	15	14	16.14	63.17	−0.172	YES
*Ac*TLP14	316	4.58	18	14	46.31	60.60	−0.018	YES
*Ac*TLP15	263	7.80	17	25	55.66	76.35	−0.075	NO
*Ac*TLP16	1056	6.60	25	112	42.18	89.82	−0.192	NO
*Ac*TLP17	183	9.12	3	15	54.35	72.62	−0.313	NO
*Ac*TLP18	287	8.46	19	26	31.35	72.37	0.014	YES
*Ac*TLP19	250	4.73	16	12	44.30	64.84	0.082	YES
*Ac*TLP20	321	5.65	16	22	45.51	61.74	−0.089	YES
*Ac*TLP21	534	4.54	35	27	44.98	51.22	−0.230	YES
*Ac*TLP22	296	4.89	20	15	36.82	67.91	0.178	YES
*Ac*TLP23	261	4.55	20	12	40.05	66.21	0.139	YES

**Table 2:** Gene duplication events for TLP family members in *A. corniculatum*.

Duplicated Gene Pairs	Ka	Ks	Ka/Ks	Duplicated Type	Divergence Time (MYA)
*Ac*TLP2 & *Ac*TLP4	1.00	0.99	1.01	Tandem	33.03
*Ac*TLP2 & *Ac*TLP3	1.00	0.99	1.01	Tandem	33.03
*Ac*TLP2 & *Ac*TLP1	1.00	1.02	0.98	Tandem	34.00
*Ac*TLP3 & *Ac*TLP4	NA	NA	NA	Tandem	NA
*Ac*TLP3 & *Ac*TLP1	0.04	0.04	0.99	Tandem	1.33
*Ac*TLP1 & *Ac*TLP4	0.04	0.04	0.98	Tandem	1.33
*Ac*TLP23 & *Ac*TLP22	0.01	0.01	1.75	Tandem	0.33
*Ac*TLP6 & *Ac*TLP21	0.27	2.54	0.10	Segmental	42.30
*Ac*TLP5 & *Ac*TLP18	0.97	1.11	0.88	Segmental	18.50

Ka: Nonsynonymous substitution rate; Ks: Synonymous substitution rate.

**Table 3:** Subcellular localization prediction by Plant-PLoc.

Query Protein	Plant-PLoc	Query Protein	Plant-PLoc
*Ac*TLP1	Chloroplast	*Ac*TLP13	Vacuole
*Ac*TLP2	Chloroplast	*Ac*TLP14	Extracellular
*Ac*TLP3	Chloroplast	*Ac*TLP15	Chloroplast
*Ac*TLP4	Chloroplast	*Ac*TLP16	Chloroplast
*Ac*TLP5	Chloroplast	*Ac*TLP17	Mitochondrion
*Ac*TLP6	Extracellular	*Ac*TLP18	Extracellular
*Ac*TLP7	Cell wall	*Ac*TLP19	Extracellular
*Ac*TLP8	Cell wall	*Ac*TLP20	Chloroplast
*Ac*TLP9	Cell wall	*Ac*TLP21	Extracellular
*Ac*TLP10	Cytoplasm	*Ac*TLP22	Extracellular
*Ac*TLP11	Extracellular	*Ac*TLP23	Extracellular
*Ac*TLP12	Extracellular		

**Table 4:** Minimal inhibitory concentration (MIC) of r*AcTLP19* against *B. cinerea* and *F. oxysporum*.

Microorganism	MIC50 (μg/mL)	
	r*Ac*TLP19	Amphotericin B
*B. cinerea*	-	4
*F. oxysporum*	-	4

“-” indicates that the MIC50 of r*Ac*TLP19 was not detected at a concentration of 128 μg/mL.

## Data Availability

The raw RNA-seq data supporting this study have been deposited in the Genome Sequence Archive (GSA) at the National Genomics Data Center (NGDC), China National Center for Bioinformation (CNCB), under BioProject accession PRJCA072618. The data are currently under review and will be publicly released upon publication. Processed expression matrices, differential expression results, and qPCR validation data are provided in the Supplementary Materials.

## Supplementary Materials

The following supporting information can be downloaded at https://www.mdpi.com/article/10.3390/genes17091023/s1, Figure S1: Plasmid map of pSmart1-*Ac*TLP19; Figure S2: CDD Search result of *Ac*TLP10 and *Ac*TLP16. (A) CDD Search result of *Ac*TLP10; (B) CDD Search result of *Ac*TLP16; Table S1: Collinearity analysis of thaumatin family members among *A. corniculatum*, *V. vinifera*, *P. trichocarpa*, and *A. thaliana*; Table S2: Sequence information of TLP family members of *A. corniculatum*. Table S3: RNA-seq profiling of transcriptional responses to pathogenic microbial infection.
