# Supplementary material for: Genome-Wide Identification and Bioinformatics Analysis of Thaumatin-like Proteins in Aegiceras corniculatum
Source: Genes (Basel). 2026 Aug 27;17(9):1023. doi: 10.3390/genes17091023 (PMC13606190; doi:10.3390/genes17091023)
Supplement: Supplementary file 1 [file genes-17-01023-s001.zip › supplementary figures.pdf]

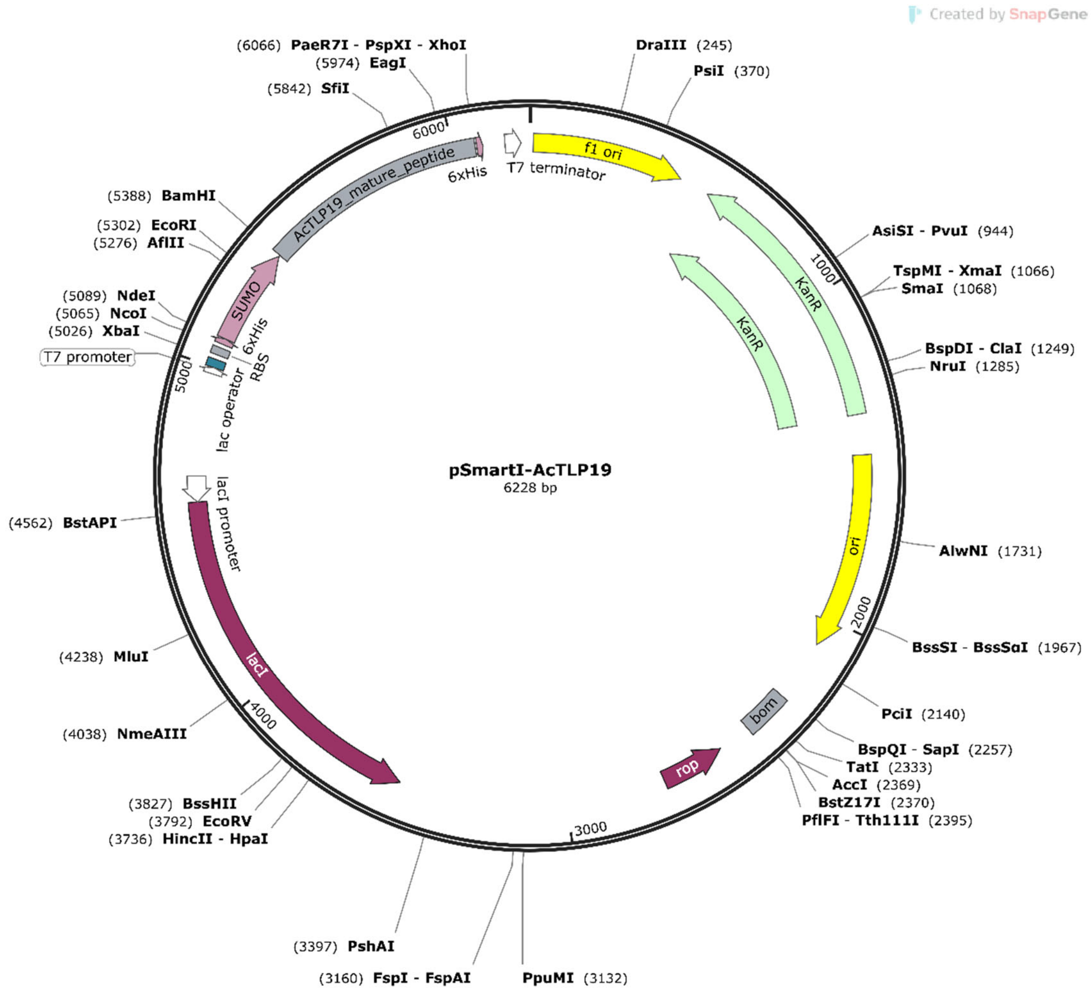

Figure S1. Plasmid map of pSmart1-*Ac*TLP19

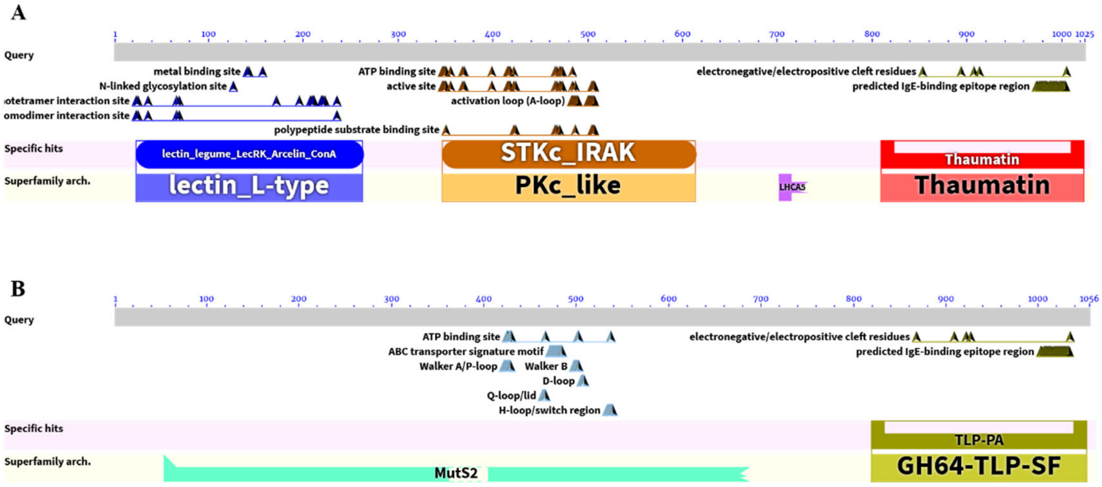

Figure S2. CDD Search result of *Ac*TLP10 and *Ac*TLP16. (A) CDD Search result of *Ac*TLP10;(B) CDD Search result of *Ac*TLP16
